# A CRISPR-Cas Cure for HIV/AIDS

**DOI:** 10.3390/ijms24021563

**Published:** 2023-01-13

**Authors:** Mouraya Hussein, Mariano A. Molina, Ben Berkhout, Elena Herrera-Carrillo

**Affiliations:** Laboratory of Experimental Virology, Department of Medical Microbiology, Amsterdam UMC, Academic Medical Center, University of Amsterdam, 1105 AZ Amsterdam, The Netherlands

**Keywords:** CRISPR-Cas, HIV, viral genes, host factors

## Abstract

Human immunodeficiency virus (HIV) infections and HIV-induced acquired immunodeficiency syndrome (AIDS) continue to represent a global health burden. There is currently no effective vaccine, nor any cure, for HIV infections; existing antiretroviral therapy can suppress viral replication, but only as long as antiviral drugs are taken. HIV infects cells of the host immune system, and it can establish a long-lived viral reservoir, which can be targeted and edited through gene therapy. Gene editing platforms based on the clustered regularly interspaced palindromic repeat-Cas system (CRISPR-Cas) have been recognized as promising tools in the development of gene therapies for HIV infections. In this review, we evaluate the current landscape of CRISPR-Cas-based therapies against HIV, with an emphasis on the infection biology of the virus as well as the activity of host restriction factors. We discuss the potential of a combined CRISPR-Cas approach that targets host and viral genes to activate antiviral host factors and inhibit viral replication simultaneously. Lastly, we focus on the challenges and potential solutions of CRISPR-Cas gene editing approaches in achieving an HIV cure.

## 1. Introduction

Human immunodeficiency virus (HIV) infections represent a major global public health burden. Approximately 38 million people are infected with HIV and 800,000 people die from HIV-related conditions every year [1]. There are currently no effective vaccines, nor is there a cure, for HIV infections [2]. Combination antiretroviral therapy (cART), the current golden standard treatment for HIV infections, is effective in controlling viral replication [2,3,4]. cART is based on a straightforward principle of targeting and inhibiting different stages of the viral replication cycle to prevent viral escape. Nevertheless, cART has multiple drawbacks [5,6]. cART must be taken by patients for the rest of their lives, it can have significant side effects, and it is still not accessible for all HIV-infected individuals worldwide. A major obstacle for HIV therapies, including cART, is the presence of long-lived and poorly understood HIV reservoirs [7,8,9]. These are cells that were once infected by replication-competent viruses, mainly CD4+ T cells, in which the HIV genome integrates into the cellular genome as a stable source for new viruses; these cells can persist over extended times. At any time, cells in the latent reservoir can become activated to start virus production, which can reignite virus replication once therapy is stopped [10]. Hence, the search for an HIV cure goes hand-in-hand with the development of strategies that trigger the eradication of viral reservoirs.

**Figure 1:**
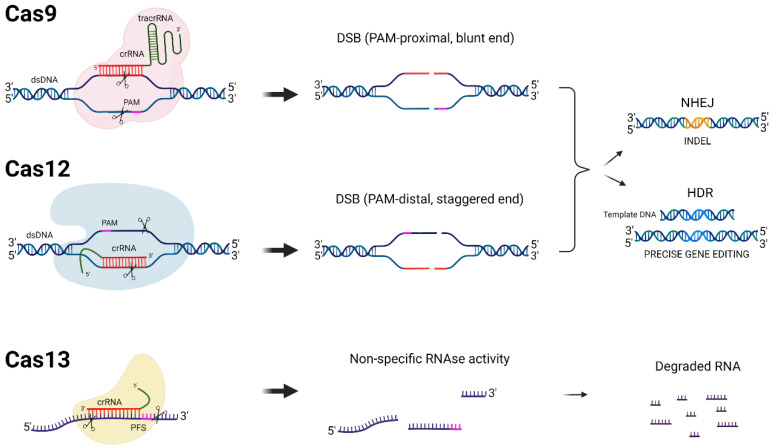
DNA- and RNA-targeting CRISPR-Cas systems. Different CRISPR-Cas proteins (Cas9, Cas12, and Cas13), their target molecules, and the cleavage mechanisms that they employ are schematically depicted. The protospacer adjacent motif (PAM) required for Cas9 and Cas12 cleavage in addition to the protospacer flanking site (PFS) required for some Cas13 orthologues are shown in pink. The crRNAs are depicted in red for each CRISPR-Cas system. When double-strand DNA breaks (DSBs) are created by Cas9 and Cas12, cellular DNA repair mechanisms are induced, either non-homologous end joining (NHEJ) or homology-directed repair (HDR). HDR requires a donor DNA template and can result in precise gene editing, while NHEJ is an error-prone repair process that introduces mutations, insertions, and deletions (INDELS, shown in orange). The Cas13 recognition and cleavage of a target RNA transcript leads to its degradation and possibly the non-specific degradation of nearby transcripts (collateral RNA cleavage).

Besides antiviral drugs, alternative methods, such as gene editing, showed encouraging results and may achieve a functional HIV cure by inactivating the integrated HIV DNA [11,12,13]. One of the most promising gene editing strategies is the clustered regularly interspaced palindromic repeat-Cas (CRISPR-Cas) platform. The CRISPR-Cas system was first identified in prokaryotes as an immune response mechanism against bacteriophage infections and invading plasmids [14,15,16]. In simple terms, the CRISPR-Cas system contains a nuclease, named Cas, which binds to a short CRISPR RNA (crRNA) that targets complementary viral DNA or RNA sequences, depending on the type of CRISPR-Cas system (Figure 1). The Cas9 and Cas12 endonucleases cleave DNA, while the Cas13 enzyme cleaves RNA. CRISPR-Cas offers a simple and efficient manner of the site-specific mutation of genomes in a wide variety of organisms [17,18]. Successful targeting requires sequence complementarity between the crRNA and the target gene. Most CRISPR-Cas systems employ a short critical 2–6 nucleotide sequence flanking the target that is named the protospacer adjacent motif (PAM) for Cas9/12 and the protospacer flanking site (PFS) for Cas13 (see Figure 1) [19,20,21].

Catalytically active Cas9 and Cas12 nucleases cut both strands of double-stranded DNA (dsDNA), producing blunt and staggered ends, respectively, and thereby activate cellular DNA repair mechanisms (Figure 1) [22,23]. In the absence of a donor template, DNA ends are repaired by an error-prone mechanism named non-homologous end joining (NHEJ). NHEJ typically leads to nucleotide insertions and deletions (INDELS), but sometimes also substitutions around the cleavage site. In the protein-coding part of a gene, INDELS may shift the open reading frame (ORF). CRISPR-Cas-based strategies have been applied as direct antivirals by mutating or excising the integrated proviral HIV DNA or indirectly by disabling viral receptors for cell entry [24,25,26]. The homology-directed repair (HDR) pathway is frequently used for gene editing carried out by donor templates [27]. Donor templates have the desired modifications flanked by segments of DNA homologous to the blunt ends of the cleaved DNA. Thus, the natural HDR DNA repair mechanism of the cell can be used to edit the genome of a target cell with high precision. Cas13 endonucleases have also been used to inhibit HIV replication (Figure 1) [28]. Cas13 leads to specific target RNA degradation without editing the genome. Note that an approach based solely on Cas13 will thus not be able to touch the integrated HIV DNA in the viral reservoir.

Recently, the Cas9 and Cas12 enzymes were modified to generate “dead Cas” or “deactivated Cas” (dCas) variants [29,30,31]. The dCas protein has the ability to bind target DNA sequences, but it cannot cleave DNA due to the loss of endonuclease cleavage activity. Instead, dCas was linked to various regulatory domains, such as transcriptional activator or repressor domains, to impose the transcriptional regulation of gene expression in a sequence-specific manner [32]. In this review, we examined the potential of the CRISPR-Cas approach targeting host and viral genes to inhibit HIV replication and ideally also to inactivate the viral reservoir.

## 2. CRISPR-Cas and HIV

**Figure 2:**
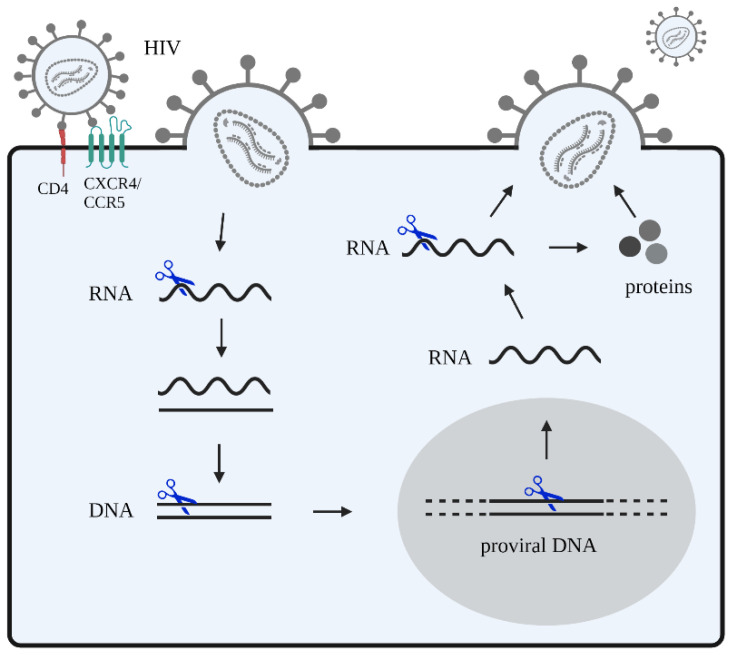
HIV attack with CRISPR-Cas-based inhibitors. HIV recognizes and binds to the host receptors CXCR4 and/or CCR5 in CD4+ T cells, and fuses with the cellular membrane. Subsequently, viral RNA is released into the cytoplasm. This viral RNA is reverse transcribed into DNA that integrates into the host genome. In the nucleus, this proviral DNA is transcribed and transported to the cytoplasm where it is translated. Viral proteins are expressed and are then packaged as well as assembled into a viral core structure that buds from infected cells and is released as new virions. CRISPR-Cas gene editing strategies (blue scissors) can be designed to target viral DNA and RNA. Other potential targets include the host DNA encoding for proteins that facilitate steps of the HIV replication cycle, such as the main CD4 receptor and co-receptors (CCR5 and CXCR4).

In terms of viral infections, and specifically HIV, the application of CRISPR-Cas editing to target and inactivate integrated viral genomes has shown positive results in several in vitro and preclinical studies [33,34,35,36]. We and others have worked extensively on the optimization of CRISPR-Cas constructs and their delivery to HIV-infected cells for virus inactivation [37,38,39,40,41]. The most direct gene therapy approach is to inactivate or remove the integrated provirus DNA from the host genome (Figure 2).

Targeting evolutionary conserved sequences minimizes viral escape [42,43]. The HIV Rev-encoding gene is an attractive target for a CRISPR-Cas-based therapy because the sequence is highly conserved among HIV subtypes [44]. By employing the CRISPR-Cas9 system to target the second exon of *Rev* in HIV latently infected cells, researchers observed a 20-fold reduction in HIV gene expression and viral production [35]. Ophinni et al. further described the importance of *Rev* as a CRISPR-Cas9 target, demonstrating that crRNA targeting Rev induced strong HIV suppression in persistently infected T cells [44]. Similarly, the CRISPR-Cas13a system, which targets RNA, was shown to inhibit HIV production and infection [28]. The authors also specifically targeted the second exon of *Rev* and found that this approach was more potent than that of other crRNAs [28]. Other CRISPR-Cas-based approaches against HIV are summarized in Table 1.

Another essential factor to consider when designing CRISPR-Cas-based gene therapies is the ability to block the viral infection of already infected cells, which is known as superinfection (SI). SI is defined as the second infection by a heterologous virus strain occurring in host cells that were previously infected and primed to exert an immunological response [63,64]. HIV has developed a mechanism to prevent SI, named superinfection resistance (SIR) [65]. SIR can be achieved through the HIV-induced downregulation of the viral entry receptor CD4 [66]. The viral proteins Nef, Env, and Vpu can regulate the expression of CD4 on infected cells, and Nef in particular downregulates the receptor [67]. Thus, to maintain SIR in HIV-infected cells upon CRISPR-Cas attack, the expression of Nef should remain intact [68,69,70]. Through the targeting of and subsequent mutational inactivation of the Rev function, HIV gene expression will be restricted to the “early” Tat and Nef proteins translated from “early” multiple-spliced transcripts. In the absence of Rev, the switch to individual and unspliced viral transcripts will not be made. Since *Rev* and *Tat* genes overlap in the HIV genome, a CRISPR-Cas approach would require the specific targeting of non-overlapping regions [71,72,73,74,75,76]. In line with this hypothesis, an early study observed that Rev-defective proviruses keep Nef intact, making these replication-defective proviruses able to exhibit SIR in infected cells and shelter them from wild-type HIV [77]. A study by Gao et al. showed a moderate decrease in the production of the HIV capsid protein CA-p24 when targeting the *Nef* gene using CRISPR-Cas12a (Table 1) [46]. Moreover, Nef counteracts the actions of host restriction factors SERINC3 and SERINC5 in order to promote HIV infection [78,79]. Therefore, additional studies involving the CRISPR-Cas targeting of Nef in the context of HIV infection are warranted.

The Tat gene has also been explored for CRISPR-Cas targeting [80,81,82]. The CRISPR-Cas9 targeting of the first exon of *Tat* induced a 53-fold reduction in CA-p24 production in latently infected cells [44]. More recently, a combinatorial CRISPR-Cas approach using three crRNAs targeting *Tat* showed the sustained inactivation of all infectious proviruses in T cell cultures [47]. These findings are in line with our previous work, in which a dual CRISPR-Cas9 approach was employed that targeted Gag and the first exon of *Tat*/*Rev*, resulting in the durable inactivation of HIV replication in T cell cultures up to 128 days and the extinction of all infectious proviruses in infected T cells [45].

The HIV genes encoding the essential Gag, Pol, and Env proteins can also be attacked by CRISPR-Cas-based approaches. Gag- and Pol-encoding regions are attractive targets for HIV antiviral strategies due to their high conservation among HIV viruses [83]. Several studies have successfully designed CRISPR-Cas9 crRNAs targeting *Gag* and *Pol* sequences and achieved the inhibition of viral production and infection [49,84]; however, this approach has not demonstrated the effective suppression of viral escape. Escape variants can arise from mutations around the cleavage site of Cas9 facilitated by the NHEJ repair of the double-strand breaks [49]. A strategy employing crRNAs targeting Gag and Pol in combination with crRNAs targeting the long terminal repeat regions (LTRs) was reported to be a more effective approach [26,36,50,51]. Likewise, Yin et al. demonstrated that saCas9 with the multiplex targeting of both the 5′-LTR and 3′-LTR elements as well as *Gag*/*Pol* efficiently inactivated proviral DNA [51]. Nevertheless, Wang et al. observed viral escape in T cell cultures when LTR and *Gag* were targeted with an spCas9 dual approach that has three target sites in HIV DNA [45]. Fan et al. described similar results by employing a dual combinatorial CRISPR-Cas12a attack against highly conserved HIV sequences [24]. The inhibition of HIV infection has also been achieved through Cas13a and Cas13d systems targeting *Gag* and *Pol* (Table 1) [28,52]. Additionally, a multiplex approach targeting four non-overlapping regions of the Pol-encoding sequences achieved the strong inhibition of HIV replication in vitro in T cell lines and primary CD4+ T cells [52].

The third gene, *Env*, is highly variable among HIV genomes when compared to *Gag* and *Pol* [85,86], which makes it a less attractive target for gene editing platforms due to the high probability of low strain coverage as well as viral escape. Nonetheless, recent data showed that combining crRNAs targeting *Gag* and *Env* can suppress viral replication [45].

**Figure 3:**
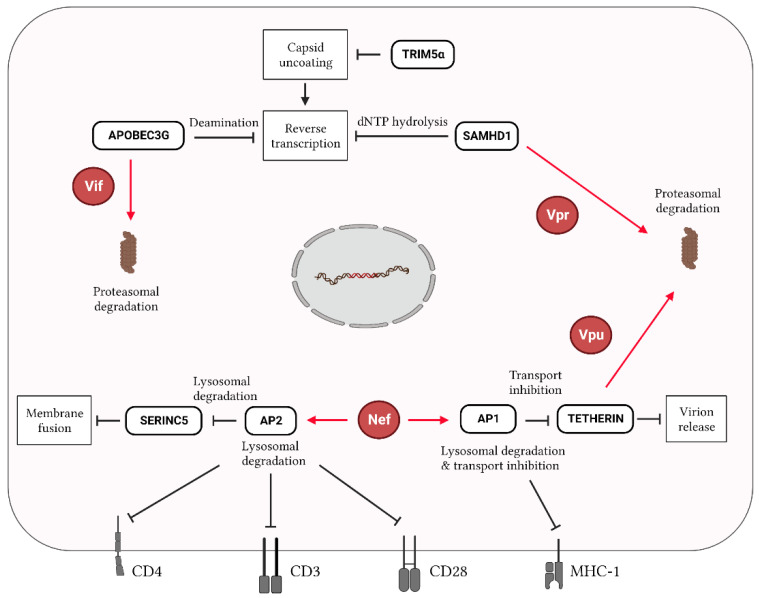
Interplay of host restriction factors and HIV proteins during infection. In HIV-infected cells, host restriction factors target several stages of the viral replication cycle. APOBEC3G, TRIM5α, and SAMHD1 restrict the reverse transcription of viral RNA, while SERINC5 and Tetherin affect HIV entry by reducing entry viral–cell membrane fusion and the HIV release of new virions, respectively. APOBEC3G, SAMHD1, and Tetherin are targeted for proteasomal degradation by Vif, Vpr and Vpu, respectively. Nef can stimulate the transcription factors AP1 and AP2 to further restrict the activity of Tetherin and SERINC5, respectively, as well as to downregulate the expression of the cell surface markers CD4, CD3, CD28, and MHC-1, thereby affecting immunity-related pathways. Red lines/arrows indicate functions exhibited by viral proteins, while black lines/arrows indicate functions exerted by host factors.

The genes encoding the accessory proteins Vif, Vpu, and Vpr could also be targeted by CRISPR-Cas approaches. These proteins have been extensively studied because they counteract the activity of cellular restriction factors and are therefore essential for viral replication [87,88,89,90,91]. One of the most studied interactions occurs between the viral Vif protein and the cellular factor APOBEC3G. APOBEC3G impedes the reverse transcription of HIV mainly through the deamination of the negative-strand DNA, resulting in hypermutations and aberrant viral transcripts (Figure 3) [92,93,94]. Vif counteracts APOBEC3G action by inducing its selective degradation [95,96]. Bogerd et al. employed CRISPR-dCas9 fused to an activation domain protein to induce the expression of the endogenous *A3B* or *A3G* promoter gene and subsequently increase APOBEC3G expression [56]. They observed the potent activation of APOBEC3G with two crRNAs, which triggered the strong inhibition of the infectivity of an HIV variant lacking the *Vif* gene [56]. Similarly, Ali et al. demonstrated that the CRISPR-Cas9 knockdown of the endogenous E3 ubiquitin ligase CHIP resulted in the stabilization of the Vif protein, which increased the ubiquitination of APOBEC3G [97]. CHIP is therefore emerging as an important restriction factor targeting Vif and as an inhibitor of HIV by promoting APOBEC3G activity.

The viral protein Vpr also interacts with multiple restriction factors: SAMHD1, the ERAD pathway, and the SLX4 endonuclease complex. SAMHD1 acts mainly as dNTPase of cellular dNTPs, depleting the dNTP pool for the viral reverse transcriptase [98,99]. In addition, SAMHD1 has RNase activity, enabling HIV RNA degradation (Figure 3) [100]. Interestingly, the CRISPR-Cas9 knockout (KO) of SAMHD1 results in a loss of HIV restriction and an increase in HIV infectivity [101,102,103]. Moreover, Vpr activates DNA damage response pathways by inducing premature cell cycle arrest at the G2 phase [104]. The interaction of Vpr with an SLX4 endonuclease complex was initially believed to be responsible for this effect [105,106,107]. An SLX4 endonuclease acts as a scaffold protein that is involved in arresting cell cycle progression until homologous recombination repairs aberrant DNA replication intermediates during DNA replication. The biological significance of this Vpr-induced G2/M arrest is to allow the processing of excess reverse transcription products, preventing their accumulation and thereby allowing the virus to remain invisible to the innate immune sensing mechanisms of the cell that can induce a potent antiviral interferon response. However, inactivating SLX4 with CRISPR-Cas9 did not abolish the DNA damage response through Vpr-induced G2 arrest [108].

Vpu plays diverse roles during HIV infection. This viral protein induces CD4 degradation, suppresses the activity of the Tetherin restriction factor, and eliminates newly made viral cDNA molecules (Figure 3) [109,110,111]. Tetherin is a type II transmembrane protein with properties that evolved evolutionary to inhibit virus release [112]. Zhang et al. employed CRISPR-dCas9 fused to the VP64 transactivation domain in combination with gRNAs targeting the *Tetherin* promoter to enhance the expression of Tetherin during HIV infection. Higher Tetherin levels triggered the inhibition of HIV production and replication, despite the presence of Vpu. Similar to Nef, Vpu contributes to SIR induction by downregulating the cell surface expression of CD4, although less efficiently than Nef [113]. Thus, it would be of interest to explore a combinatorial therapy by targeting HIV while maintaining Vpu expression. Even though several HIV restriction factors have been identified, it remains poorly understood as to whether a CRISPR-Cas approach targeting these HIV restriction factors would result in the long-term suppression of HIV replication; more studies evaluating these strategies are needed. CRISPR-Cas tools also allow for the identification of novel host restriction factors and their interactions with accessory viral proteins, which provide an array of new potential targets for HIV antiviral strategies [114,115].

Alternatively, one could use CRISPR-Cas to mediate the integration of antiviral genes of interest. Nahmad et al. described a dual adeno-associated virus (AAV) CRISPR–saCas9-mediated integration of antibody genes into the Ig loci of primary human B cells to express anti-HIV broadly neutralizing antibodies (bNAbs) in vivo [62,116]. Using this method, the authors achieved high titers of anti-HIV bNAbs that neutralize infection-competent pseudoviruses in mice [62,116].

## 3. Challenges and Potential Solutions in Designing a CRISPR-Cas-Based Therapy for HIV

CRISPR-Cas provides a simple and easily scalable platform for targeting a specific site in the genome, and it can be applied to target HIV genes and boost host restrictions factors [117]. Attempts to achieve an HIV cure through CRISPR-Cas gene editing have been promising, but viral escape, immunogenicity, off-target effects, and efficient delivery in patients remain major challenges in developing a successful curative therapy.

### 3.1. Single versus Combinatorial CRISPR-Cas Approaches

**Figure 4:**
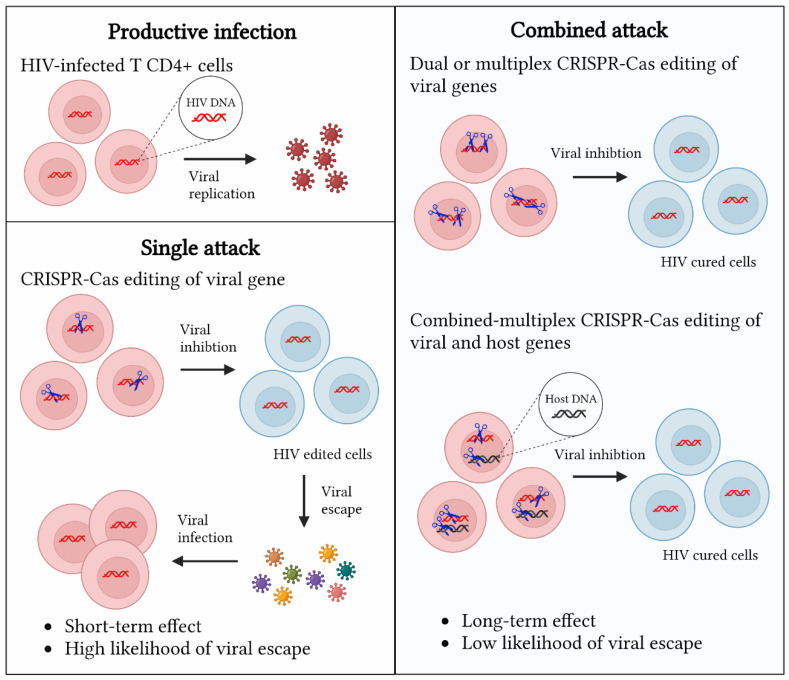
Comparison of CRISPR-Cas editing platforms for gene therapy of HIV infections. CRISPR-Cas editing platforms (blue scissors) against HIV can be classified into three categories: the single targeting of HIV genes, the dual or multiplex targeting of HIV genes, and a combined targeting of HIV genes and host DNA to boost the activity restriction factors.

Current CRISPR-Cas approaches include the single and multiplex targeting of viral genes (Figure 4). Single approaches consist of targeting viral genes with a single crRNA. They generally exhibit a high likelihood of viral escape, as the DNA repair mechanism NHEJ induces viral mutations that trigger viral escape [49]. The more durable suppression of HIV can be achieved through multiplex approaches targeting HIV with two or more crRNAs against a single gene or multiple genes [42,45]. These multiplex approaches have long-lasting effects and a low likelihood of viral escape [47]. Alternatively, one could design a combinatorial approach by targeting both viral and host genes that encode important cofactors, and additional regimens could be considered, such as the addition of a gene encoding virus-neutralizing antibodies. Aside from multiplexing, we may also be able to minimize viral escape through the selection of a proper Cas system. Unlike Cas9, Cas12a performs multiple rounds of editing because the cleavage site is distantly located downstream of the PAM sequence, and thus is less likely mutated by the cellular DNA repair machinery, which will allow for multiple rounds of editing and likely the introduction of more gross mutations that would prevent viral escape [46].

### 3.2. Off-Target Effects

Developing a CRISPR-Cas combined strategy against HIV also means that it will be important to evaluate the potential challenges to therapy. Potential off-target effects of Cas nucleases in the host genome complicate the development of safe gene therapies [118,119,120]. We should be aware that there is a risk of gene editing outside the target locus. Nonetheless, these off-target effects can possibly be predicted and limited through several strategies [121,122,123], such as the modification of crRNAs or Cas proteins to boost the specificity of the editing event [123]. There is also the risk of unwanted large chromosomal deletions that can potentially lead to unwanted gene silencing, the removal of a tumor suppressor gene, or the activation of a proto-oncogene that could trigger uncontrolled cell growth and oncogenesis. This warrants an intense safety screen for anti-HIV CRISPR approaches in HIV-infected individuals.

### 3.3. Delivery Methods

We realize that a successful HIV cure will be a daunting task. The major obstacle to an HIV cure is the presence of a multifaceted HIV reservoir. These cells were infected with replication-competent viruses, mainly CD4+ T cells, and are able to persist over time under therapy as well as reignite active virus replication once therapy is stopped. The heterogeneous nature of a reservoir challenges the success of a cure approach unless we can efficiently target all of the different components of an HIV reservoir. The targeting of HIV reservoir cells could be facilitated by guiding therapeutic interventions using selective markers for the target cells. For instance, we believe that CD32a-positive cells form a major component of the reservoir [124,125], but other cell types will likely contribute to the reservoir. Thus, clearing integrated HIV DNA from all infected cells and tissues remains very difficult if not impossible.

Viral delivery methods are widely used for CRISPR-Cas therapies [24,126,127]. Adeno-associated viruses (AAVs) are currently the leading in vivo delivery system of CRISPR components because of low immunogenicity, high target cell specificity, and limited integration into the genome of the host cell. However, the packaging capacity is limited (4.2 kb), and the genetic material encoding the most frequently used spCas9 (~4 kb) leaves little space for the necessary regulatory elements. Different strategies have been proposed to reduce the size of the cargo, such as the splitting of spCas9 into two fragments that are introduced into the cell via different AAV vectors, where they can recombine to form an active editing enzyme. However, such intricate strategies usually come with significantly reduced editing efficiency [128].

Lentiviral vectors (LVs) are able to infect dividing as well as non-dividing cells and have a higher cloning capacity (~8.5 kb) than AAVs, making them a suitable platform for the delivery of CRISPR-Cas components with a single viral vector [129,130]. LVs are currently the most widely used viral vectors for clinical gene therapy applications when long-lasting transgene expression is required because LVs integrate into the host genome [131]. Unlike retroviral vectors (RVs), LVs have never caused leukemia in gene therapy trials due to integration-related oncogenesis effects [131]. However, the prolonged expression of the Cas protein by stably integrated LVs may increase the risk of off-target effects, and therefore safety features should be explored further.

Non-integrating LV systems have been proposed as a transient delivery method. Non-integrating LVs for the transient delivery of CRISPR components were designed via the mutation of the viral integrase gene or the long terminal repeats (LTRs) that carry important integration signals [132,133]. Alternatively, one could use non-viral delivery methods such as lipid-based ribonucleoproteins (RNPs) [134,135], polymer-based RNPs [136], or inorganic materials for CRISPR-Cas delivery [137].

### 3.4. Immunogenicity

The immunogenicity of CRISPR-Cas gene editing platforms should also be considered for the translation of a combinatorial approach into the clinic. Since the CRISPR-Cas system is of bacterial origin, an immune reaction against it is likely to occur when it is administered in vivo. Reports demonstrate that people can possess pre-existing immunity against certain Cas proteins that can frustrate the delivery [138,139,140]. This problem could be avoided by selecting a non-immunogenic Cas system or by the use of immune-suppressive drugs in parallel with CRISPR-Cas-based therapy. Alternatively, antibody responses can be partly mitigated through the mRNA delivery of Cas instead of RNPs or by the encapsulation of RNPs into lipids or polymers to shield the immunogenic protein from antibodies.

Overall, several challenges remain for the implementation of CRISPR-Cas-based treatments against HIV. Rational and systematic designs of multiplex approaches based on knowledge of the host restriction pathways as well as viral genes may further support the long-term suppression of HIV infections.

## 4. Conclusions

CRISPR-Cas gene editing techniques have been widely used to target DNA in eukaryotic cells and represent a promising strategy against HIV infections and for achieving an HIV cure. Current and novel insights into HIV infection biology may aid in improving such CRISPR-Cas-based gene therapy strategies. Combined approaches that simultaneously target viral genes and boost the activity of host restriction factors can be proposed to realize long-lasting antiviral effects in an infected individual. Long-term in vitro and in vivo infection experiments with distinct Cas systems, in addition to different crRNAs and delivery systems, are needed to further evaluate the efficiency and safety of this technology. Analyses of the potential for viral escape, off-target effects, and the immunogenicity of CRISPR-Cas gene editing strategies will further assist in the selection of suitable systems and approaches that can be tested in appropriate preclinical and clinical trials. Overall, these studies will hopefully bring us closer to achieving an effective antiviral therapy against HIV that could help millions of people worldwide currently suffering from the disease.

## Figures and Tables

**Table 1:** Summary of CRISPR-Cas gene editing approaches against HIV.

Source	Target	System	Delivery ^a^	Platform ^b^	Findings	References
Virus	Rev	Cas9, Cas13a	LV	Single, dual, and multiplex	Inactivation of latent proviral DNA, suppression of viral RNA, and HIV elimination	[28,35,44,45]
	Nef	Cas12a	LV	Single	Modest inhibition of viral production	[46]
	Tat	Cas9, Cas12a	LV	Single, dual, and multiplex	Inhibition of viral production and viral escape, HIV elimination	[24,44,45,47,48]
	Gag	Cas9, Cas12a, Cas13a, and Cas 13d	LV, AAV	Single, dual, and multiplex	Inhibition of viral production, inactivation of latent proviral DNA, and HIV elimination *	[24,26,28,45,49,50,51,52]
	Pol	Cas9, Cas13a, and Cas13d	LV, AAV	Single, dual	Inhibition of viral production	[28,49,50,52,53]
	LTR	Cas9, Cas12a	LV	Single, dual, and multiplex	Inhibition of viral production and replication *	[24,45,54,55]
	Env	Cas9	LV	Dual	Inhibition of viral production, HIV elimination	[45]
Host	A3G, A3B	Cas9	LV	Single, dual	Activation of host restriction factors	[56]
	Tetherin promoter	Cas9	LV	Single	Inhibition of viral production and replication	[57]
	CCR5	Cas9	AAV	Single, dual	Gene editing of receptor, inhibition of viral replication, and HIV resistance	[58,59,60]
	CXCR4	Cas9	LV	Dual	Gene editing of receptor, inhibition of viral replication, and HIV resistance	[61]
	B cells	Cas9	AAV	Single	Insertion and induction of the synthetic anti-HIV broadly neutralizing antibody 3BNC117	[62]

^a^. Lentiviral (LV) or adeno-associated vectors (AAVs). ^b^. Use of one crRNA (single), two crRNAs (dual), or more than two crRNAs (multiplex). * Viral escape detected.

## Data Availability

No new data were created or analyzed in this study. Data sharing is not applicable to this article.
